# World Wide Web Resources on Obstetrical and Gynecological Infections

**DOI:** 10.1155/2007/29232

**Published:** 2007-09-18

**Authors:** Stavros Athanasiou, Eleni Pitsouni, Christos Iavazzo, Eirinaios M. Karamanis, Matthew E. Falagas

**Affiliations:** ^1^Alfa Institute of Biomedical Sciences, 9 Neapoleos Street, 15123 Marousi, Athens, Greece; ^2^1st Department of Obstetrics and Gynecology, Athens University School of Medicine, Athens, Greece; ^3^Department of Medicine, Tufts University School of Medicine, Boston, MA 20110, USA

## Abstract

Modern information and communications technology has provided medical students and practitioners around the world with a new, valuable, and easy-to-use way to retrieve potentially useful information. Using previously described by our research group methodology, we generated a list of 50 Internet resources in the field of obstetrical and gynecological infections. We believe that the availability of such a list will help in the education of students and clinicians interested in obstetrical and gynecological infections.

Nowadays the need for obtaining updated medical information is essential to doctors in practice. The fast progress in medical science is a fact that cannot be ignored. This statement also applies to the field of infectious diseases, including obstetrical and gynecological infections. For example, the recent introduction of the vaccine for human papilloma virus (HPV) represents a major advance in the battle against cervical cancer. The interest on obstetrical infections is of paramount importance because of the effects such infections have on pregnant women as well as on fetuses and newborns. Therefore, consultation of appropriate educational resources on diagnostic tests, treatment, and management options, in general, on obstetrical and gynecological infections is useful to practitioners, especially obstetricians and gynecologists.

Under these circumstances, the World Wide Web may be seen as a very useful educational tool for practitioners and students. It provides quick and easy access to information of different kinds, including medical knowledge. In many occasions, information provided by World Wide Web sources is updated frequently (monthly, weekly, or even daily). Although, there are, many popular and well-known sites that can provide useful medical information, a significant number of them are not widely in use.

The purpose of this contribution is to compile a list of World Wide Web sites that include information regarding obstetrical and gynecological infections see Table 1. All of the sites that we included in our list are free-accessed and in English. We did not include in our list of World Wide Web sites well-known worldwide valuable resources such as PubMed. The methodology we used in this paper has been described in previous relevant contributions of our team [1–3]. An effort was made to gather as many sites as possible referring to obstetrical and gynecological infections. However, we recognize that some sites that offer valuable information on this issue were overlooked. We think that the list of World Wide Web sites may be found useful by practicing obstetricians and gynecologists around the world, especially those clinicians with Internet access practicing in developing countries.

## Figures and Tables

**Table 1:** World Wide Web resources on obstetrical and gynecological infections.

Website title	Website address	Comments
WHO	http://www.who.int/topics/en	Links and guidelines for surveillance, prevention, of management and a treatment of ob/gyn infections

Centers for disease control and prevention	http://www.cdc.gov/std	Information about prevention, surveillance, and treatment of STD

European center for disease prevention and control	http://www.eurosurveillance.org/index-02.asp	Surveillance of communicable infections across Europe

The American College of Obstetrics and Gynecologists	http://www.acog.org	Information about infections and many useful links

Royal College of Obstetricians and Gynaecologists	http://www.rcog.org.uk	Clinical guidance on ob/gyn infections

The Society of Obstetrics and Gynecologists of Canada	http://www.sogc.org/guidelines/index_e.asp	Clinical practice guidelines

Obgyn.net	http://www.obgyn.net/search/search.asp	Information about infections and useful links

Lincoln center	http://www.lincolncenterobgyn.com/links.htm	Links to ob/gyn sites

Infectious diseases in obstetrics and gynaecology	http://www.hindawi.com/journals/idog	An open access journal with special interest in ob/gyn infections (free access)

The internet journal of gynaecology and obstetrics	http://www.ispub.com/ostia/index.php?xmlFilePath=journals/ijgo/front.xml	Online free-access journal (free access)

Contemporary ob/gyn	http://www.contemporaryobgyn.net/obgyn	Full articles about ob/gyn infections

British Columbia Ministry of Health	http://www.bchealthguide.org/womens.stm	Online guide of women's health including useful links

Pan American Health Organization	http://www.paho.org	Links to sites about the prevention and management of communicable diseases and their surveillance

National Library for Health	http://www.library.nhs.uk/?autoLogin=0	Evidence-based reviews, guidance, and specialist libraries

University of California, Department of Medicine	http://medicine.ucsf.edu/resources/guidelines	Links to sites that provide clinical guidelines

The University of Iowa Libraries	http://www.lib.uiowa.edu/hardin/md/obgyn.html	Links to images, symptoms, and treatment for ob/gyn infections

North Carolina, School of Medicine	http://www.med.unc.edu/obgyn	Updated information for obstetricians and gynaecologists

Kirksville College of Osteopathic Medicine, Faculty of Microbiology and Immunology	http://www.kcom.edu/faculty/chamberlain/std.htm	Symptoms, diagnosis, and treatment of STD

University of Alberta	http://www.obgyn.med.ualberta.ca/pdf/0405Talks/Turnell/Peri_Infs_2004.pdf	Lecture slides about perinatal infections

University of Tennessee, Department of Pediatrics	http://www.utmem.edu/pediatrics/residency/Library/perinatal_infx2006.ppt	PowerPoint presentation about the diagnosis and therapy of perinatal infections

Geneva Foundation for Medical Education and Research	http://www.gfmer.ch/Guidelines/Obstetrics_gynecology_guidelines.php	Links to prevalence symptomatology, images, and treatment guidelines about ob/gyn infections

The Royal Women's Hospital (RWH)	http://rwh.org.au/nets/handbook/index.cfm?doc_id=4972#rub_intro	Congenital infections information

National Women's Health at Auckland City Hospital	http://www.adhb.govt.nz/newborn/Guidelines/Infection/CongenitalInfection.htm	Guidelines for investigation of suspected cases

The Hospital for Sick Children	http://www.motherisk.org/prof/infectiousDiseases.jsp	Issues about infectious diseases during pregnancy

Karolinska Institute	http://www.mic.ki.se/MEDIMAGES.html#GynecologyObstetrics	Useful links to images, illustrations, and case presentations

Guttmacher Institute Practical	http://www.guttmacher.org	Issues and reports from all around the world

National institute of Allergy and Infectious Diseases	http://www3.niaid.nih.gov/research/topics	Overviews about the ob/gyn infections

Clinical Microbiology Reviews	http://cmr.asm.org	Full-text articles

Chlamydiae.com	http://www.chlamydiae.com	A site dedicated to chlamydia

Candida corporation	http://www.candida.com	A site dedicated to candida

Mycoplasma.com	http://www.mycoplasma.com	Links to sites dedicated to mycoplasma

Ureaplasma.com	http://www.ureaplasma.com	Links to sites dedicated to mycoplasma

International Herpes Virus Forum	http://www.ihmf.org	Slide lectures, diagnostic atlas, and management guidance for herpes infection

Virology. net	http://www.virology.net	Information and links about viruses including those causing ob/gyn infections

Wong's Virology	http://virology-online.com/presentations/index.htm	PowerPoint presentations about congenital viral infections, Herpes viruses, and more ob/gyn related viruses

American Pregnancy Association	http://www.americanpregnancy.org/pregnancycomplications/index.htm	General information about infections during pregnancy

National Network of STD/HIV Prevention Training Centers	http://depts.washington.edu/nnptc/index.html	STD clinical training

The National Women's Health Information Center	http://www.4woman.gov	Immunization, symptoms, and treatment guidance

Family Health Information	http://www.fhi.org/training/en/modules/STD/tools.htm	PowerPoint presentation about STDs with extra slide corresponding narrative

National network for immunization information	http://www.immunizationinfo.org	Guidance for immunization for pregnant women, prevention of HPV, etc.

British Association for Sexual Health and HIV	http://www.bashh.orgn	Site dedicated to sexual health and HIV

Globalrph.com	http://www.globalrph.com	Clinician guide to drug therapy

Organization of Teratology Information	http://otispregnancy.org/otis_fact_sheets.asp	Specialists fact sheets

Perinatology.com	http://www.perinatology.com/exposures/infectionlist.htm	Information about infections, symptoms, treatment options, etc., during pregnancy; useful links

Thefetus.net	http://www.thefetus.net/listing.php?id=1718	Information about infections, symptoms, ultrasound images, treatment options, etc., during pregnancy

International Birth Defects Information System	http://www.ibis-birthdefects.org/start/infect.htm	Useful links to images, diagnostic tests and treatment options about congenital infections

Wikipedia.org	http://en.wikipedia.org/wiki/Main_Page	Free online encyclopedia

General Practice Notebook	http://www.gpnotebook.co.uk/homepage.cfm	Interactive information about congenital and ob/gyn infections

Netterimages.com	http://www.netterimages.com/image	Netter medical illustration site

AccessMyLibrary	http://www.accessmylibrary.com	Online news, research, and information, free registration
